# In vitro and in vivo analyses on anti-NSCLC activity of apatinib: rediscovery of a new drug target V600E mutation

**DOI:** 10.1186/s12935-022-02723-7

**Published:** 2023-02-09

**Authors:** Jiani Chen, Jingwen Zhai, Mingming Li, Shiyi Liu, Xiaobin Gong, Hongyu Yu, Hua Wei, Wansheng Chen

**Affiliations:** 1 Medical Guarantee Center, Second Affiliated Hospital of Naval Medical University, Shanghai, 200003 China; 2 Department of Pathology, Second Affiliated Hospital of Naval Medical University, Shanghai, 200003 China

**Keywords:** Apatinib, BRAF V600E, Non-small-cell lung cancer (NSCLC), HPLC-MS/MS, P-gp, CYP3A4, CYP2D6

## Abstract

**Background:**

Apatinib (YN968D1) is the first small-molecule-targeting drug with anti-tumor activity created in China for the treatment of advanced gastric cancer (GC) and hepatocellular carcinoma (HCC). It showed significant variation in the efficacy for treating cancers, including advanced non-squamous non-small-cell lung cancer (NSCLC). Whether its efficacy could be optimized by subgrouping patients with certain genetic variation remains elusive.

**Methods:**

Here, we firstly used kinase screening to identify any possible target of apatinib against 138 kinases. The effects of apatinib on proliferation rates, cell cycle, cell apoptosis, and cell migration on cancer cell lines were analyzed; the in vitro potential pathways of apatinib on cancer cell lines were screened. The effect of apatinib on mouse cancer models in vivo was also analyzed.

**Results:**

Based on HCC364 cells with BRAF V600E mutation, we have shown that apatinib could inhibit their growth, migration, cell cycle, and induce their apoptosis. Based on mice with transplanted HCC364 cells, we have also shown that apatinib could inhibit the tumor growth. Based on immunohistochemistry, we have demonstrated that apatinib could suppress the phosphorylation of mitogen-activated protein kinase/extracellular signal-regulated kinase and extracellular regulated protein kinases. This may account at least part of the apatinib’s inhibitory effect on HCC364 cancer cells.

**Conclusions:**

BRAF V600E protein kinase is a target of apatinib by kinase screening. We have demonstrated that apatinib can effectively inhibit tumor cells with BRAF V600E mutation by in vitro and in vivo experiments. Our results have demonstrated that targeting BRAF V600E mutation, apatinib appears to be effective and safe for treating NSCLC and possibly other cancers with the same mutation.

**Supplementary Information:**

The online version contains supplementary material available at 10.1186/s12935-022-02723-7.

## Background

Lung cancer is the most common cancer worldwide and its incidence increased rapidly in China during the past decades. It is also the leading cause of cancer-related mortality with a 5-year survival rate of 15% [1]. Chemotherapy is the dominant treatment means of lung cancer, which is applied for more than 80% of patients alone or in combination with radiotherapy [2]. Non-small-cell lung cancer (NSCLC) accounts for more than 85% of all lung cancers [3]. In the last few years, many new target therapies have been approved by FDA in treatment of NSCLC. Refining the targets of these drugs may greatly increase their efficacy.

It is well-known that 91% of anti-tumor drug susceptibility is closely related to proto-oncogenes [4], and nearly 80% of these proto-oncogenes encode protein kinases. A protein kinase is an enzyme that modifies other proteins by phosphorylation on serine, threonine or tyrosine residues of a protein, dysregulation, which is closely related to human oncogenesis [5]. Both the number of protein kinases and the ways of modification may contribute to the diversity of protein kinase dysregulation. Protein kinase constitutes 2% of human genome sequencing, and 500 ones have been structurally recognized [6, 7]. And they can be modified by over-expression, relocation, and fusion, point mutations, or dysregulation of upstream signaling [5, 8, 9].

Even if the mutation rates of driver genes, including BRAF and MET are low [10, 11], the development and application of targeted therapy drugs is still quite important, which can be used to improve the survival outcomes of NSCLC patients due to the base number of NSCLC patients is large.

It is reported that BRAF mutation rate in NSCLC is up to 3.5–4%, and BRAF V600E mutation is found to be the mosot frequent BRAF mutation in lung cancer, accounting for roughly 50% of these BRAF-mutant NSCLC cases [10]. It was reported that NSCLC patients with BRAF V600E-mutant tumors had a poorer prognosis compared with the non-V600E ones [12]. In NSCLC, the biologic behavior of BRAF-mutated lung tumors tend to be more aggressive [13].

Apatinib (YN968D1) is the first generation of oral antiangiogenesis drug created in China widely used in the treatment of various solid tumors, including advanced gastric cancer (GC) and hepatocellular carcinoma (HCC) with safety and efficacy. Apatinib is a multi-targeted tyrosine kinase inhibitor, which mainly targets vascular endothelial growth factor receptor-2 (VEGFR2) kinase as well as platelet-derived growth factor receptor β (PDGFR-β) and c-kit; it plays the anti-tumor role by inhibiting neovascularization [14–16]. It can significantly prolong the overall survival (OS) and progression-free survival (PFS) for advanced GC, with reliable safety feature [14–16]. However, the efficacy of VEGFR-based treatment differs significantly among individuals [17], which might be a result of other undiscovered anti-tumor mechanisms or genetic variations amongst individuals. However, there is no report to evaluate its efficacy and safety in patients with NSCLC. In recent research, an increasing number of valuable real-life evidence support that apatinib has been proven to be effective against NSCLC.

Consistently, a phase II clinical trial using apatinib to treat NSCLC in Asia has showed prolonged mPFS (4.7 m vs 1.9 m, P < 0.001) and better response rate (RR) (12.2% vs 0%, P = 0.0158), compared with the control group [18]. This indicated that apatinib could provide further benefits in specific subpopulations. On the other hand, sorafenib (an inhibitor of VEGFR, PDGFR, and c-KIT), another small-molecule-targeting drug with the similar targets to those of apatinib, did not show an efficacy as good as apatinib. Amongst Caucasians, the RR and mPFS of the experimental group were 4.9% and 2.8 months respectively [19]. These results suggested that apatinib should be further investigated on its effect and mechanism on NSCLC.

In this study, we screend the potential kinase targets of apatinib in vitro and followed by in vivo validating experiments on mice to provide scientific evidence for clinical applications. This study can provide new insights into the the strategy of NSCLC and promote individualized apatinib medication for rational clinical application in NSCLC patients.

## Materials and methods

### Cell and animal models

Melanoma cell lines: A375, A375R, A2058, WM-115, SK-MEL-28, WM-3152, WM-2664, WM-1789, SK-MEL-2; colorectal cancer cell lines: HT-29, Colo-205, Colo-205R, NCI-H508, LoVo; lung cancer cell lines: HCC364, HCC364R, NCI-H1666, NCI-H1755, NCI-H2087, NCI-H460, HCC827. Among them, HCC364 cells were purchased from Sur biological (Shanghai), and those resistant to Vemurafenib and HCC364R and Colo-205R were constructed by the laboratory in the Department of Pharmacy of Shanghai Changzheng Hospital, and the rest were purchased from the Cell Bank of the Institute of Life Sciences of the Chinese Academy of Sciences (Shanghai).

Balb/c nude mouse (Nanjing University-Nanjing Institute of Biomedical Research, Nanjing, license number: SCXK (Su) 2015-0001). The animal experiment was approved by Ethics Committee of Changzheng Hospital of Shanghai. Throughout the experiment, all operations were based on the corresponding requirements of AAALAC.

### Standards and reagents

Apatinib (purity ≥ 98%, Jiangsu Hengrui Pharmaceutical Co., Ltd.), vemurafenib (purity ≥ 98%, Selleckchem, USA), vemurafenib raw materials (purity ≥ 98%, Biedue, Shanghai). RPMI-1640 culture medium (Hyclone, USA), fetal bovine serum (Invitrogen, USA), double antibody (Invitrogen, USA), 0.25% trypsin (Invitrogen, USA). CCK8 Assay Kit (Tongren Chemical, Japan), Annexin V-FITC/PI Apoptosis Detection Kit (BD, USA), DMSO (Sigma, USA). BCA Protein Assay Kit (Pierce, USA), M-PER Cell Extraction Reagent (Pierce, USA). MEK/p-MEK antibody, ERK/p-ERK antibody (Abeam, USA), hypersensitive ECL chemiluminescence detection kit (Thermo, USA), polyvinylidene difluoride membrane (PVDF membrane) (Millipore, USA), Matrigel (BD, USA). sodium carboxymethyl cellulose (CMC-Na) (Sinopharm Group, Beijing), hydroxypropyl cellulose (Klucel LF) (Changwei Medicine, Shanghai), DMSO (GmbH, Germany), Tris-Tris (Hydroxymethyl) aminomethane, hydrazone red S, Tween-20, and β-mercaptoethanol were all chemical analysis reagents purchased from Amresco, USA.

### Kinase screening

LanthaScreen kinase activity assay and Z’-LYTETM platform of Life Technologies Corporation were used to screen the inhibitory activity of 100 nM apatinib against 138 protein kinases, including BRAF and AKT. The inhibitory rates higher than 40% were considered as good inhibitory activity.

### Cell culturing conditions

Corresponding culture medium of each cell line was selected, in which secondary-antibody (100 U/mL penicillin and 100 pg/mL streptomycin), and the culturing conditions were 37 °C, 5% CO_2_ and 95% relative humidity. When the cell density reached 70%, the subculture was carried out by trypsinization. Fresh complete culture medium was adopted for replacement every other day.

### The effect of apatinib on proliferation rates of A375, HCC364, Colo-205 cell lines

This experiment was divided into one control group and 7 drug-administered groups with different concentrations (25, 50, 100, 200, 400, 800 and 1600 nM). The control group was treated with an equal volume of DMSO. The activity test was carried out 48 h later by using the CCK-8 kit with triplicate analyses for each cell line. Each analysis was averaged from measurement at different locations of the culturing well. The inhibitory rate (IC_50_) of each cell line was calculated based on absorbance of 450 nm light by Graphpad (San Diego, CA, US).

### The effect of apatinib on cell cycle and apoptosis of A375, HCC364, Colo-205 cell lines

This experiment consisted of four groups: a blank control group, a vehicle control group, a control group with 20 nM vemurafenib and a treatment group with 20 nM apatinib. Cell cycle and apoptosis analyses were both completed 48 h after administration of drugs or vehicle solution, and they were completed by published method and Annexin V-FITC/PI kit respectively. Triplicate analyses were done for each cell line.

### The effect of apatinib on cell migration of A375, HCC364, Colo-205 cell lines

A375, HCC364 and Colo-205 cells in the logarithmic phase were prepared, cell suspension was prepared by trypsinization, cell pellet/precipitation was collected by centrifugation, and cell density was adjusted to 1 × 10^5^/mL cells with RPMI-1640 complete culture medium containing 10% FBS. These cells were inoculated into a 24-well cell culture plate (50 μl of Matrigel at a concentration of 10 ug/mL was pre-added, and it was gently shaken to make cells evenly distributed without bubble emerging, overnight in the refrigerator at 4 °C, and 500 μL of cell suspension was added to each well. The plate was gently shaken to make cells evenly distributed. When these cells were cultured for 24 h (37 °C and 5% CO_2_), cell scratch wound assay was conducted with a sterile 200 μL tip, then these cells were washed twice with serum-free medium, and finally 2 mL of 1% serum was added. Drugs or vehicle solution were/was added to each cell culture medium as required and they were grouped; these cells were cultured under normal conditions, and photographed by microscope after 72 h. This experiment was performed with 3 biological replicates (n = 3).

### In vitro pathway analyses of A375, HCC364, Colo-205 cell lines

The cells were divided into five groups: a control group (vehicle solution), a control group with vemurafenib (20 nM), and three treatment groups with apatinib (10 nM, 20 nM and 50 nM).

A375, HCC364 and Colo-205 cells in the logarithmic phase were prepared, cell suspension was prepared by trypsinization, and the viable cell count was performed by trypan blue dyeing. The cells were collected by centrifugation at 1000 × *g* for 2 min. The RPMI-1640 culture medium containing 10% FBS was used to resuspend the cells, and cell concentration was adjusted to 1 × 10^5^ /mL. The cells were inoculated into a 6-well culture plate (2 mL per well), overnight at 37 °C and 5% CO_2_. 2 mL of fresh complete culture medium was adopted for replacement per well. According to the final concentration of drugs, vemurafenib and apatinib was added to two groups respectively. The control group was added with an equal volume of DMSO to the drug intervention groups. And then the cells were cultured for 48 h under normal conditions and corresponding phosphorylation levels of the proteins in the regulatory pathway was detected.

The assay was performed 48 h after administration. The total protein was extracted by using M-PER, quantified by BCA kit with15 μg added to each well, electrophoresed in 11% SDS-PAGE, and transferred to a nitrocellulose membrane. And then, it was blocked with BSA for 2 h; the PVDF membrane was shaken slowly on the shaking table at room temperature. The cells were washed twice by TBST to remove the excess BSA; and the primary antibody diluted in a certain ratio was added: MEK (1:300), p-MEK (1:500), ERK (1:600), p-ERK (1:350), VEGFR (1:300), p-IVEGFR (1:500), AKT (1:400) and p-AKT (1:400), overnight in the refrigerator at 4 °C. The membrane was washed three times with TBST, and the primary antibody was removed from the membrane surface, followed by the secondary antibody (rabbit anti-mouse, 1:2000) added, and they were reacted at room temperature for 2 h. The luminescent liquid was configured according to the ECL kit instructions, dropped onto the PVDF membrane, and cells were imaged with X-ray film. The target band was scanned, and its optical density was analyzed. The degree of protein phosphorylation was analyzed and the total protein content was used as reference. This experiment was performed with 3 biological replicates (n = 3).

### Preparation of working solution and experimental animals

Apatinib working solution was prepared by slowly adding apatinib powder to a 0.5% CMC-Na solution and dispersing it to make solutions at concentrations of 1.6, 3.25 and 5 mg/mL. Vemurafenib working solution was prepared by slowly adding Vemurafenib powder to a 0.2% Klucel LF solution and uniformly dispersing it to make a solution at concentration of 6.25 mg/mL.

Female Balb/c nude mice (6 weeks old, 20 ± 2 g), reared for 7 days to adapt to the environment (temperature: 24 ± 2 °C, relative humidity: 60 ± 10%, day and night (light and dark) alternate time: 12 h/12 h, free diet, free drinking water) after purchase. Tumor cells in the logarithmic growth phase were amplified and collected, and cell suspension was prepared: Matrigel = 1:1 cell mixture; the tumor cells were inoculated subcutaneously into the right lower back of the mice, and the number of cells inoculated was 5.0 × 10^6^ volume of 0.1 mL.

When the tumors were grown to 100–200 mm^3^, 30 animals were selected according to their body weight and tumor volume, which were randomly divided into 5 groups, each group with 6 rats, and administered by intragastric administration as follow: (1) 5%CMC-Na, qd, 2 mL/100 g; (2) 6.25 mg/mL Vemurafenib, bid, 2 mL/100 g; (3) 1.6 mg/mL Apatinib, qd, 2 mL/100 g; (4) 3.25 mg/mL Apatinib, qd, 2 mL/100 g; (5) 5 mg/mL Apatinib, qd, 2 mL/100 g. The HCC364 model was administered for 26 days, the A375 and Colo-205 models were administered for 21 days. All animals were euthanized within 2 h when the experiment ended.

### Statistical analyses

Differences between groups were analyzed by SPSS 19.0, and one-way ANOVA was used in combination with Dunnett (when variance homogeneity) or Dunnett's T3 (when variance was not uniform). P < 0.05 was considered to indicate a significant difference, P < 0.01 was considered to indicate a highly significant difference.

## Results

### Screening for target kinase

The abnormal expression of protein kinase in tumor cells has been found to be closely related to tumor invasion, migration and angiogenesis, which has become an important target of anti-tumor drugs. In order to find the de novel target of apatinib, we screened it against a series of carcinoma cells. They were from different cancer types. Some of them are BRAF V600E positive and some of them are negative. The inhibitory activity of 100 nM apatinib against 138 kinases was screened. Amongst them, the inhibitory rates of 15 kinases such as KIT Y823D and BRAF V600E were higher than 40% (Additional file 1: Table S1). In addition, BRAF V600E was closely related to tumor development. Therefore, we can produce reliable evidence supporting that BRAF V600E is a potential target for NSCLC.

### In vitro analyses of the effect of apatinib on tumor cell lines

**Fig. 1:**
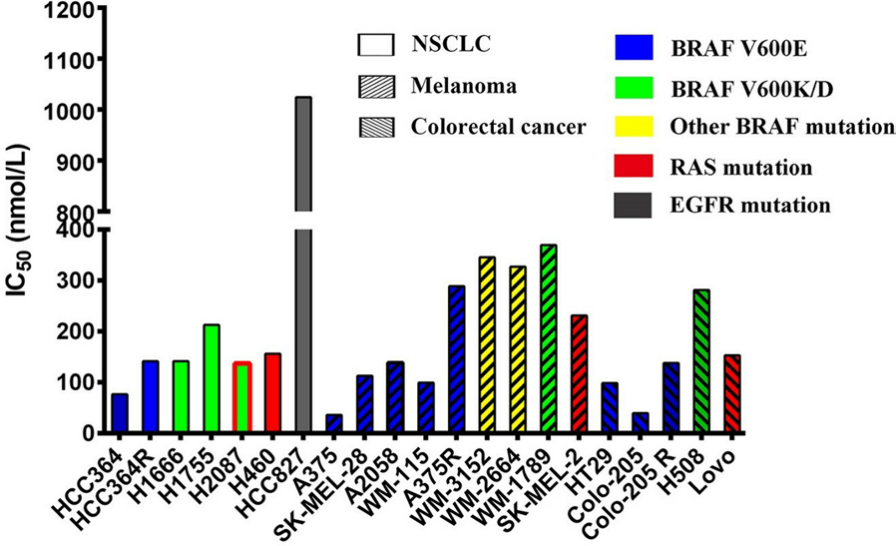
The IC_50_ of apatinib against 21 cell lines. Data were expressed as mean ± SD. All experiments were performed with 3 biological replicates

Firstly, the CCK8 method was used to detect the inhibitory effect of apatinib on the proliferation of 21 tumor cells (Fig. 1). It showed that the inhibitory activity of apatinib on cell proliferation was concentration-dependent, and it had good inhibitory activity against tumor cell lines with BRAF V600E or RAS mutations. However, its inhibitory activity against HCC827 cells with EGFR mutation was weak compared with BRAF V600E or RAS mutations (P < 0.01).

**Fig. 2:**
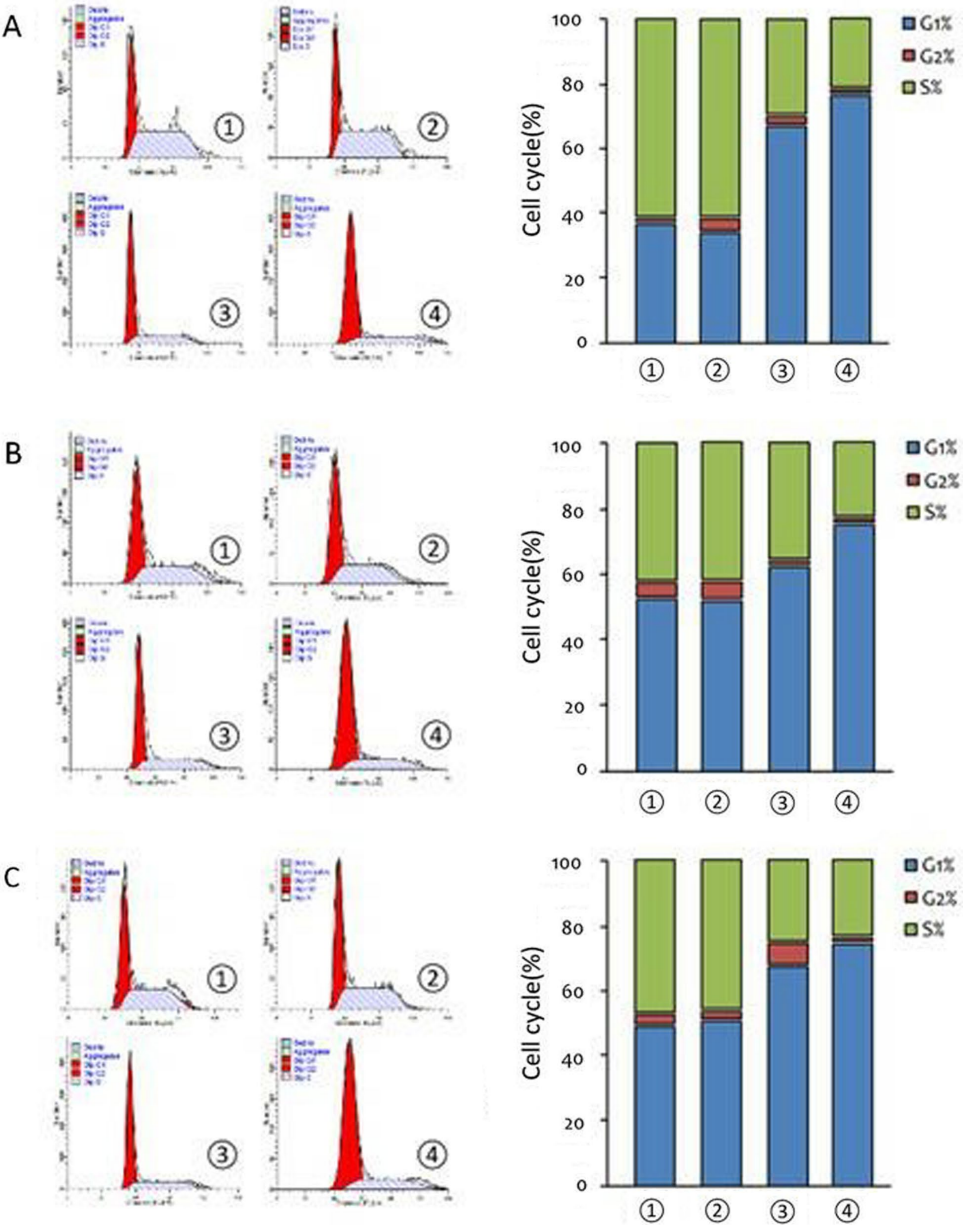
Cell cycle experiments. **A**, **B**, and **C** are cell cycle detection data of A375, HCC364 and Colo-205, respectively. The left is the detection of raw data; the abscissa is PI; the ordinate is the number of cells. The right is the cell cycle data mapping, the abscissa is the cell grouping; the ordinate is the proportion of cells in each phase. Data were expressed as mean ± variance, and all experiments were performed with 3 biological replicates (n = 3). ① control group (without treatment), ② vehicle control group, ③ vemurafenib control group (20 nM), ④ apatinib treatment group (20 nM)

Subsequently, the inhibitory effect of apatinib was further studied on three cell lines: A375, HCC364 and Colo-205. Being added with 20 nM of apatinib or vemurafenib for 48 h, the three cell lines were significantly blocked at the G1 phase. The effect of apatinib treatment group is more prominent on blocking cell cycle at the G1 phase than other groups (Fig. 2).

**Fig. 3:**
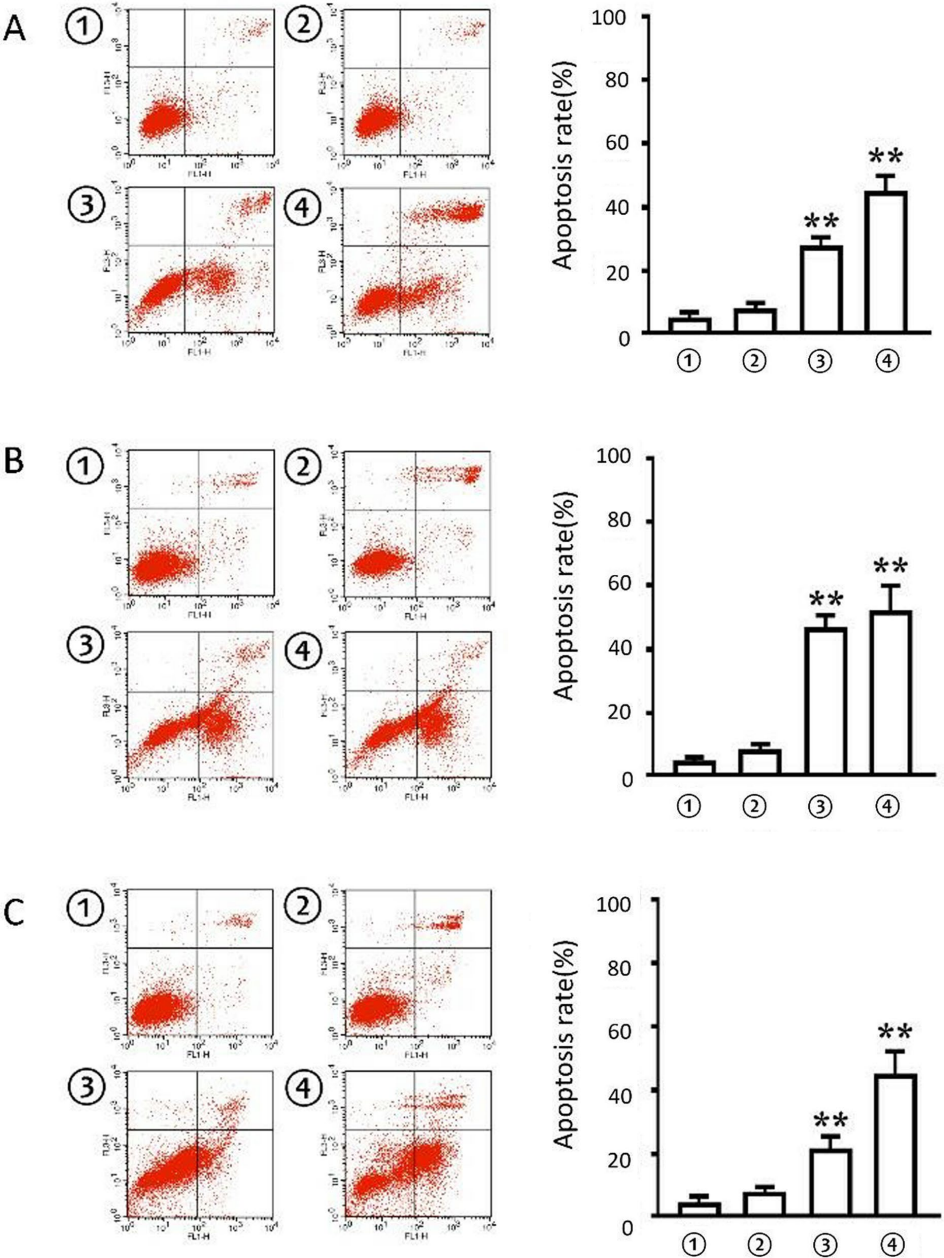
Cell apoptosis experiments. **A**, **B**, and **C** are the apoptosis data of A375, HCC364 and Colo-205, respectively. The left side is the raw data, the abscissa is FITC; the ordinate is PI; the lower-right quadrant is pre-apoptosis, and the upper-right is late apoptosis. The right is the cell cycle data mapping, the abscissa is the cell grouping, the ordinate is the proportion of apoptotic cells, and the apoptotic value is the sum of the pre-apoptosis and the late apoptosis. Data were expressed as mean ± variance, and all experiments were performed with 3 biological replicates (n = 3). ^**^P < 0.01.^*^P < 0.05 vs cell control or vehicle control group. ① control group (without treatment), ② vehicle control group, ③ vemurafenib control group (20 nM), ④ apartinibweretreatment group (20 nM)

**Fig. 4:**
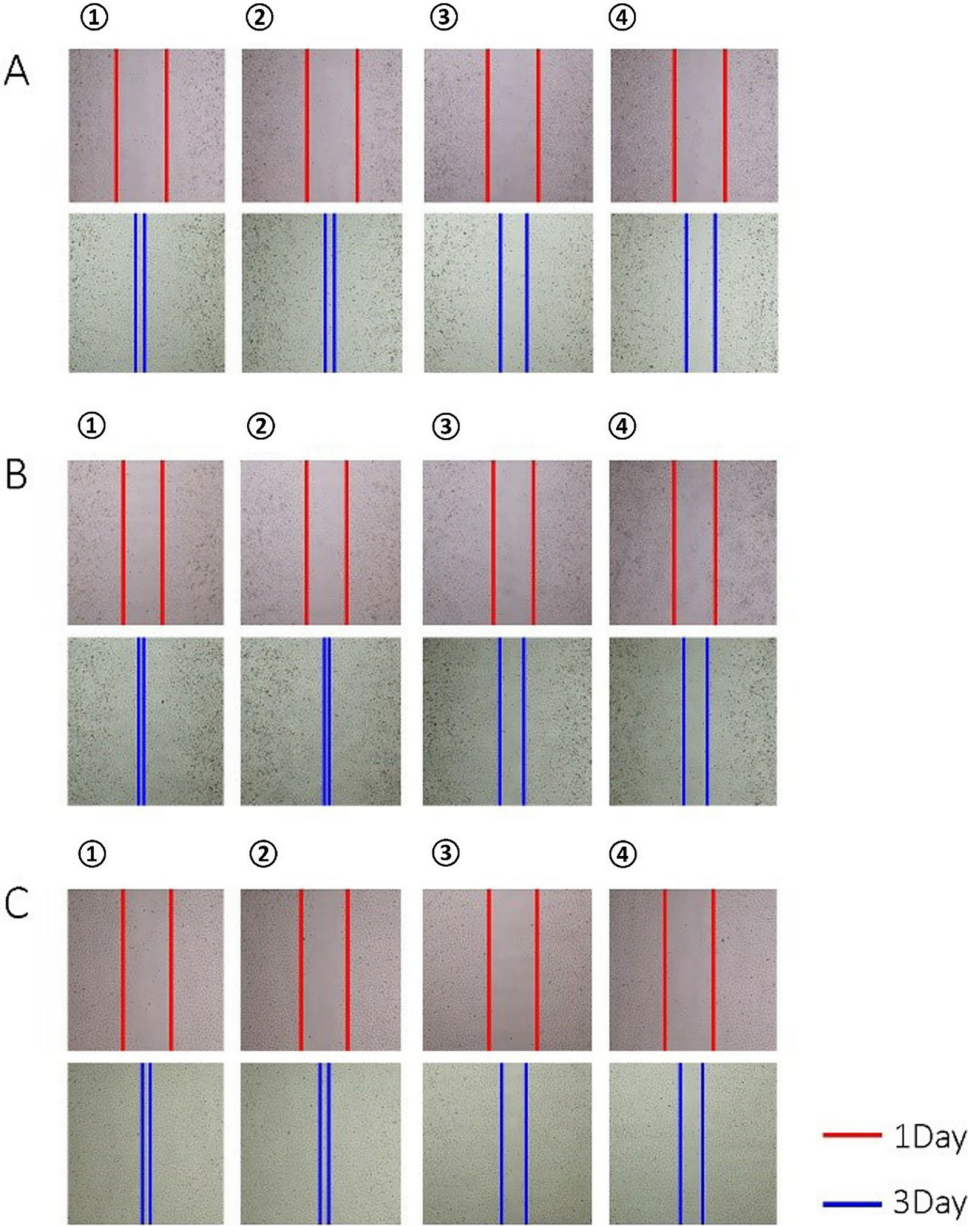
Cell migration assays. **A**, **B**, and **C** are the results of cell migration assays of A375, HCC364, and Colo-205, respectively. The above figure shows the pre-dose photographs. The first row in each result shows the photos taken at the uniform scribing position before administration and the second row shows the photos taken after 48 h of administration. Distance between the two lines reflects cell migration ability. Five groups of visual fields were randomly selected from each group of cells. ① control group (without treatment), ② vehicle control group, ③ vemurafenib control group (20 nM), ④ apatinib treatment group (20 nM)

The effect of apatinib on apoptosis of A375, HCC364 and Colo-205 cells is shown in Fig. 3. The apoptosis rates of A375, HCC364 and Colo-205 in the 20 nM apatinib treatment group were 40.90 ± 3.34%, 46.95 ± 7.12%, 42.68 ± 5.83%, respectively. For the 20 nM vemurafenib group, those were 24.34 ± 2.02%, 43.83 ± 3.49%, 19.56 ± 4.68% respectively. They were all higher than the two control groups whose apoptosis rates were 4.80 ± 0.04%, 4.30 ± 0.02%, 2.99 ± 0.04% or 8.58 ± 0.04%, 8.54 ± 0.05%, and 7.06 ± 0.03% respectively (P < 0.01). It seemed that apatinib also had a stronger effect on inducing tumor cell apoptosis than vemurafenib. In addition, apatinib could also reduce the migration rates of A375, HCC364 and Colo-205 cell lines with the same degree as vemurafenib’s (Fig. 4).

**Fig. 5:**
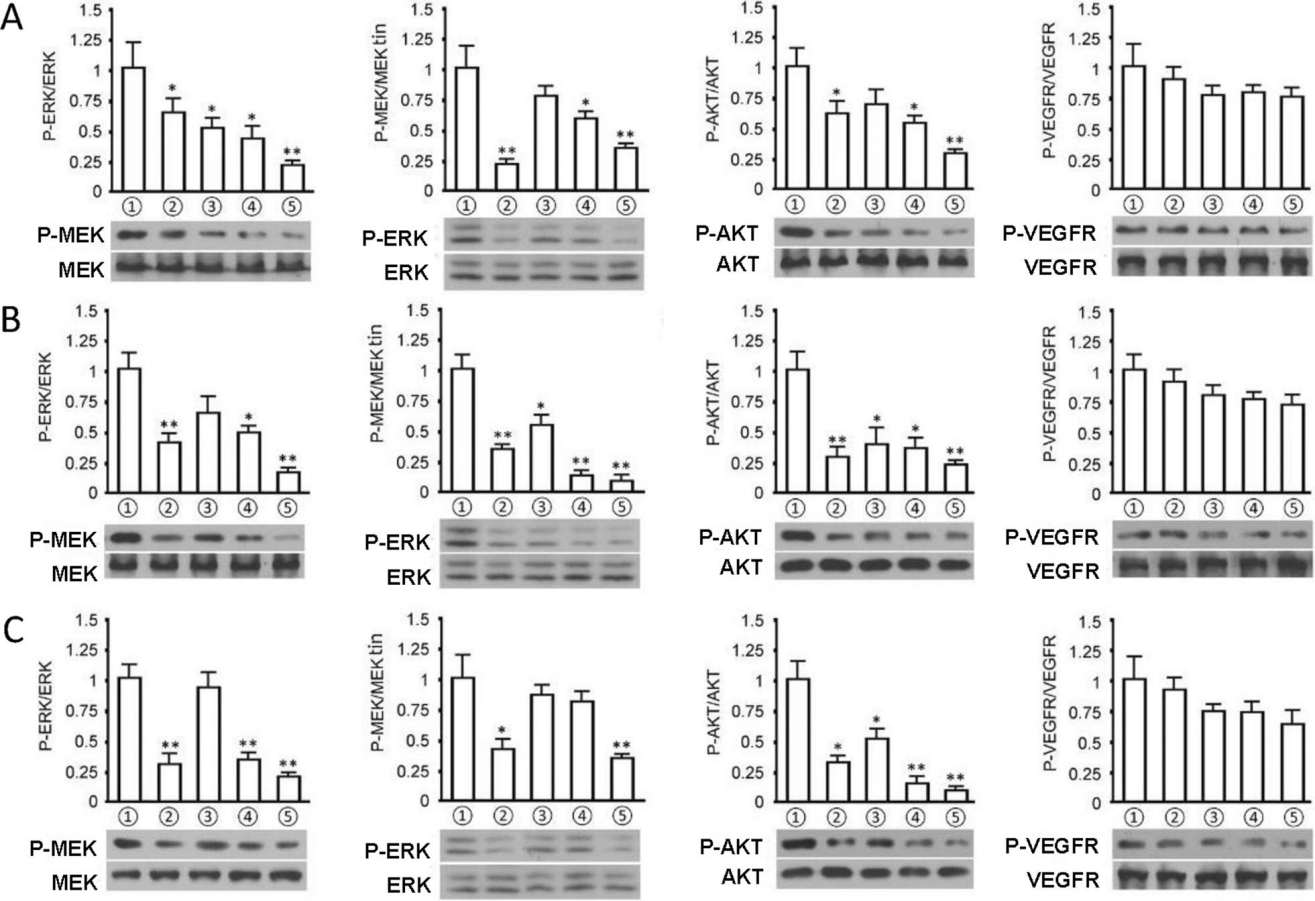
Immunohistochemistry analyses based on cellular proteins. **A**, **B**, and **C** are A375, HCC364, and Colo-205 cell protein assay data, respectively. The above figure shows the relative optical density of each group of cells. The figure below shows the target band after exposure of the target protein. The degree of protein phosphorylation is expressed as the ratio of phosphorylated protein to total protein. Three biological replicates (n = 3) were set in the experiment, and the data were expressed as mean ± SD, ^*^P < 0.05.^**^P < 0.01 vs. cell control group. ① control group, ② vemurafenib control group, ③ apatinib treatment group (10 nM); ④ apatinib treatment group (20 nM), ⑤ apatinib treatment group (50 nM)

Finally, the potential signal pathways of apatinib were screened (Fig. 5). The results showed that apatinib treatment groups with concentrations of 10 nM, 20 nM and 50 nM could significantly inhibit the phosphorylation of downstream MEK, ERK and AKT proteins of BRAF pathway, and the inhibitory effect was gradient-dependent. In addition, the control group with 20 nM of vemurafenib could also significantly inhibit the phosphorylation of MEK, ERK and AKT in each repeated experiment, compared with the controls (P < 0.05). The treatment groups with apatinib at different concentrations and with 20 nM vemurafenib for 48 h could inhibit the phosphorylation of VEGFR in these tumor cell lines; however, there was no significant difference compared with the controls (P > 0.05).

### In vivo analyses of the effect of apatinib on tumor-bearing mice

**Fig. 6:**
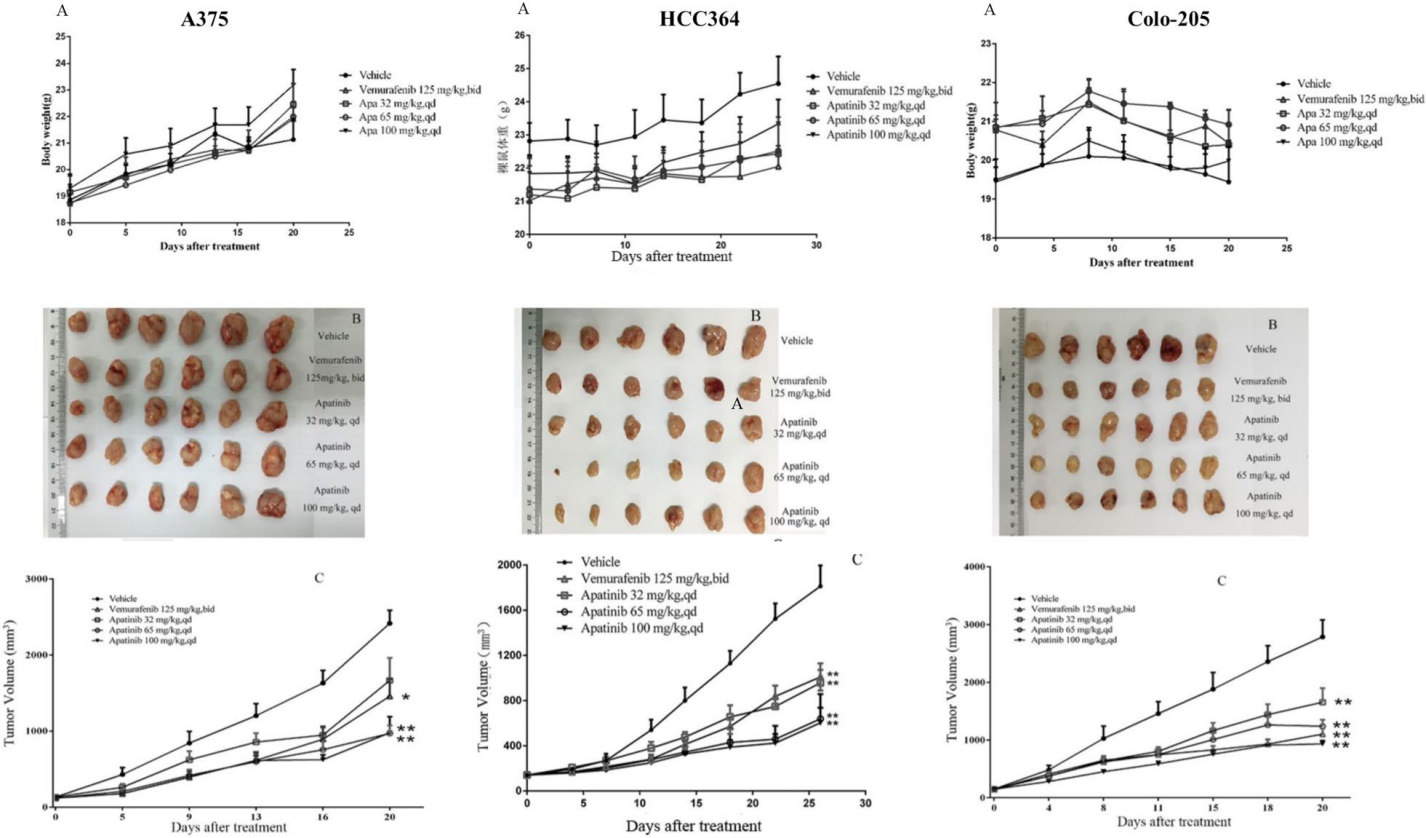
Tumor suppression effects of apatinib. From left to right, data were acquired from mice transplanted with A375, HCC364 and Colo-205 cell linesm, respectively. The fisrt row illustrates the effect of apatinib on the body weight of mice. The second and the third rows illustrated the effect of apatinib on the tumor sizes (n = 6)

**Fig. 7:**
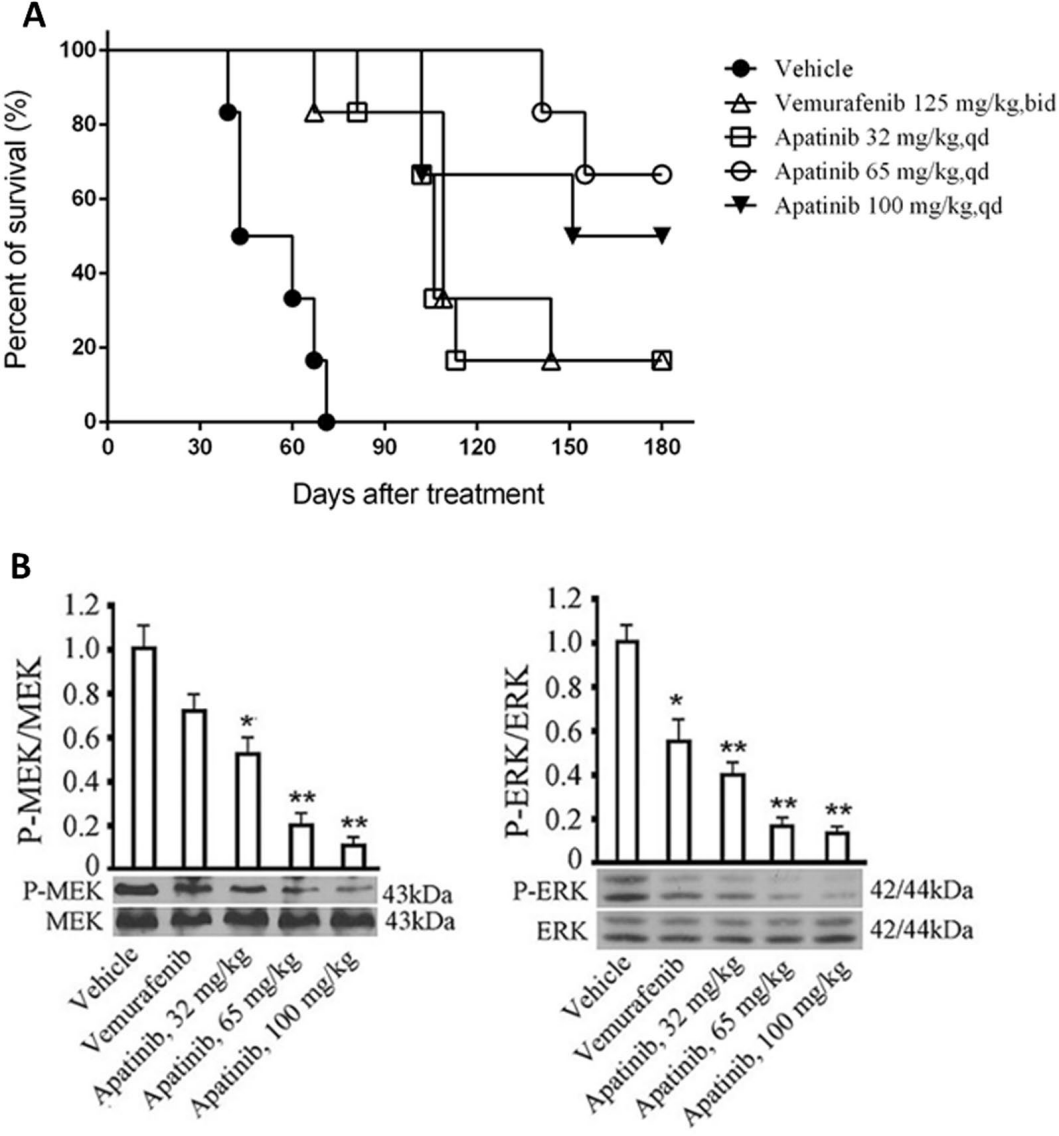
Beneficial effect of apatinib HCC364 mice and possible cellular mechanism. **A** Kaplan–Meier survival curve of HCC364 nude mice. **B** Inhibition of apatinib on tumor tissue signaling pathway in HCC364 nude mice; the figure above shows the comparison between the groups of protein phosphorylation rates; the figure below shows the scan of the target bands. 6 nude mice per group (n = 6), the data is expressed as ^*^P < 0.05, ^**^P < 0.01 (vs. vehicle group)

The body weight of nude mice was not significantly changed after being administered with apatinib at different concentrations (P > 0.05), but the tumor volume growth was obviously inhibited after being administered wtih apatinib and vemurafenib (P < 0.05) (Table 1, Fig. 6, and Additional file 2: Fig. S1). After 26 days of continuous administration, the mice were sacrificed. Then, the tumor tissues were removed to examine the phosphorylation levels of MEK and ERK (Fig. 7B). The phosphorylated levels of MEK and ERK were both significantly decreased after administration of apatinib (P < 0.05); however, the phosphorylated levels of MEK of vemurafenib was not significantly affected (P > 0.05).

**Table 1:** Inhibitory effect of apatinib on growth rate of nude mice transplanted with A375, HCC 364 or Colo-205 cells

Cell line	Drug	Doses (mg/kg)	BW (g)		V (mm^3^)		RTV	TGI (%)
			d0	dn	d0	dn		
A375	Control	–	18.7 ± 0.5	21.7 ± 0.5	134.2 ± 11.6	2416.1 ± 173.4	18.5 ± 2.0	–
	Vemurafenib	125, bid	18.7 ± 0.3	21.9 ± 0.4	120.1 ± 9.9	1458.6 ± 228.6^a^	12.1 ± 1.5^a^	34.85
	Apatinib	32, qd	19.2 ± 0.6	22.5 ± 0.8	133.7 ± 12.6	1665.3 ± 297.2	12.1 ± 1.3^a^	34.50
	Apatinib	65, qd	18.8 ± 0.3	22.0 ± 0.6	121.3 ± 10.8	971.7 ± 102.7^b^	8.1 ± 0.7^b^	56.26
	Apatinib	100, qd	19.3 ± 0.5	23.2 ± 0.6	121.6 ± 10.1	978.5 ± 211.5^b^	7.8 ± 1.0^b^	57.90
HCC364	Control	–	22.1 ± 0.5	24.6 ± 0.8	139.6 ± 10.3	1813.3 ± 183.1	13.4 ± 1.7	–
	Vemurafenib	125, bid	21.0 ± 1.1	22.1 ± 0.6	141.2 ± 14.9	1009.2 ± 120.0^b^	7.6 ± 1.1^b^	48.73
	Apatinib	32, qd	21.2 ± 1.1	22.4 ± 1.1	140.8 ± 14.1	958.2 ± 114.2^b^	6.9 ± 0.7^b^	51.58
	Apatinib	65, qd	21.4 ± 0.9	22.5 ± 0.8	139.8 ± 10.3	639.3 ± 217.9^b^	4.7 ± 1.4^b^	70.19
	Apatinib	100, qd	21.8 ± 0.6	23.4 ± 0.7	140.7 ± 11.5	604.3 ± 132.2^b^	4.2 ± 0.8^b^	72.49
Colo-205	Control	–	19.5 ± 0.5	19.4 ± 0.9	149.3 ± 7.8	2784.1 ± 295.9	18.5 ± 2.1	–
	Vemurafenib	125, bid	20.8 ± 0.3	20.4 ± 0.5	144.0 ± 5.0	1101.2 ± 98.8^b^	7.6 ± 0.5^b^	58.82
	Apatinib	32, qd	20.8 ± 0.6	20.4 ± 0.9	148.4 ± 8.8	1654.0 ± 243.2^b^	11.2 ± 1.7^b^	39.37
	Apatinib	65, qd	20.9 ± 0.3	20.9 ± 0.4	146.1 ± 7.5	1238.0 ± 111.6^b^	8.4 ± 0.4^b^	54.49
	Apatinib	100, qd	19.4 ± 0.4	20.0 ± 0.5	148.4 ± 7.9	932.2 ± 57.2^b^	6.4 ± 0.5^b^	65.63

^a^P < 0.05

^b^P < 0.01

The effect of apatinib on the survival of tumor-bearing nude mice is shown in Fig. 7A. There were significant differences between the vemurafenib or apatinib groups and the vehicle control group (Log-rank P < 0.0001). Both the survival rate of mice administrated with apatinib and vemurafenib were significantly higher than than the controls. More interestingly, the survival rate of mice administrated with apatinib at the dose of 65 mg/kg was significantly higher compared with the control group with vemurafenib at the clinical dose (Log-rank P = 0.0380).

## Discussion

Apatinib (YN968D1), is a multi-target tyrosine kinase inhibitor, similar to vatalinib (PKT787), which can effectively inhibit VEGFR, PDGFR and c-Kit receptor kinases [20]. Apatinib may also be a multi-target tyrosine kinase inhibitor, since the chemical structure of a drug defines its function. To explore the potential anti-tumor targets of apatinib, we have examined all tyrosine kinases and important silk/threonine kinases associated with lung cancer including Akt 1-3, FRAP1, PIK3C2A, PIK3C2B, BRAF, BRAF V600E and RAF1 in the Life platform [21]. Amongst them, we found that BRAF V600E mutation was the most affected protein kinase by apatinib. This was followed by cells with other mutations within MAPK signaling pathway and upstream RAS mutations. However, it exerted a weak inhibitory influence on the EGFR-mutant lung cancer cell line, HCC827 (IC_50_ = 1024 nM). Therefore, we speculated that it was not suitable for treating patients with EGFR mutations. This result might, to some extent, explain the reason for the substantial intra-patient difference of the efficacy of apatinib. It was also indicated that BRAF V600E mutation was a potential anti-tumor target of apatinib for the first time [22].

BRAF protein is a critical kinase in the mitogen-activated protein kinase (MAPK) signaling pathway, encoded by the BRAF gene, activating downstream MEK and ERK signal pathway-related proteins and can regulate cell proliferation, differentiation and apoptosis [23, 24]. BRAF is an important proto-oncogene in humans, whose tumor mutation rate is approximately 8%. BRAF mutations are common in melanoma, ovarian cancer, thyroid cancer, and colorectal cancer. The BRAF V600E mutation accounts for about 50% of BRAF mutations. The BRAF V600E-mutated tumor cell has a higher kinase activity compared with the wild type [25], which promotes proliferation and inhibits apoptosis [26], finally reducing the sensitivity to patients receiving chemotherapy drugs [22, 27].

Consistent with the bimolecular significance of BRAF protein and its V600E mutation in carcinogenesis, several drugs targeting BRAF V600E mutations have been approved or under development to treat melanoma, ovarian cancer, thyroid cancer and colorectal cancer [28]. Nonetheless, there are only clinical studies have provide proof that BRAF V600E mutation is also a potential marker of target therapy in improving the prognosis for NSCLC patients. It has been shown that vemurafenib or apatinib could significantly inhibit tumor growth in NSCLC patients with BRAF V600E mutantion who did not respond to conventional chemotherapy regimens [29, 30]. Around the same time, a phase II clinical trial showed that vemurafenib had a better RR (42%) in advanced NSCLC patients with BRAF V600E mutation than various conventional chemotherapy regimens [31].

In this study, the inhibitory activity of apatinib against BRAF protein kinase was verified by both in vitro and in vivo experiments. The effects of apatinib on cells with BRAF V600E mutation included promotion in cell apoptosis, inhibition in migration and invasion, suppression in cell cycle at the G1 phase, and inhibition in phosphorylation levels of MEK, ERK, and Akt. However, it showed no significant effect on the phosphorylation level of VEGFR2.

It was speculated that drugs targeting BRAF V600E mutation do not have cross-resistance. In one clinical study, bronchial adenocarcinoma patients with BRAF V600E mutation were firstly treated with vemurafenib; however, these patients had a progression. They were then treated with docetaxel chemotherapy for 4 months, followed by targeted therapy with dabrafenib. After these treatments, abdominal pain disappeared, the ascites was significantly reduced, and the prognosis was improved [29]. Consistently, apatinib also had good inhibitory activity against HCC364R, Colo-205R and A375R cells induced by vemurafenib, suggesting that after resistance to vemurafenib, patients may still benefit from apatinib.

With the promising results from our cell experiment, we further investigated the inhibitory effect of apatinib at different concentrations in a mouse model transplanted with NSCLC tumor. In a phase III clinical trial of apatinib in patients with advanced NSq NCLC, the clinically acceptable doses of apatinib were 250, 500 and 750 mg/days, respectively; for mice, the equivalent dose were 32, 65 and 100 mg/kg, respectively. We found that groups with medium and high doses of apatinib had good inhibitory activity on the growth of different tumor cells in vivo. In addition, the inhibitory effects of apatinib at the medium and high doses were better than vemurafenib.

Finally, we investigated the possible inhibitory mechanism of apatinib. ERK is a signaling protein in the MAPK signaling pathway. It is located downstream of RAF and MEK and is activated by phosphorylation, which is of great significance in proliferation and differentiation of tumor cells. In this experiment, the phosphorylation levels of MEK and ERK1/2 protein in each tumor tissue were detected by Western Blotting method, and the phosphorylation rates of each group were compared. The results showed that the phosphorylated levles of MEK and ERK in the tumor tissues was significantly inhibited (P < 0.05) after administration of apatinib, and the inhibition was dose-dependent. This result is consistent with the trend of our in vitro experimental results. NSCLC cells with BRAF V600E mutation were significantly inhibited by apatinib and vemurafenib, which could inhibit the phosphorylation of signaling protein downstream BRAF signaling pathway. Finally, the growth of tumor was significantly inhibited after administration of apatinib or Vemurafenib. This indicated that drug resistance was not developed against apatinib.

## Limitation

This study demonstrated the important role of apatinib in BRAF V600E mutation for NSCLC. Nevertheless, This study still has several limitations. First, the mechanism of apatinib inhibiting HCC364 cells has not been completely elucidated since only in vitro and in vivo experiments were examined. Second, rescue studies are required to clarify the biological mechanism that apatinib could suppress the activity of BRAF V600E.

## Conclusions

We have explored that the BRAF V600E protein kinase is a target of Apatinib by kinase screening. We have also demonstrated that apatinib can effectively inhibit tumor with BRAF V600E mutation through in vitro and in vivo experiments with promising results. Our results have demonstrated that targeting BRAF V600E mutation, apatinib appears to be effective and safe for treating NSCLC and possibly other cancers with the same mutation.

## Supplementary Information

**Additional file 1: Table S1.** The inhibitory activity data of apatinib against each kinase.

**Additional file 2: Figure S1.** Tumor suppression effects of apatinib. From left to right, data were acquired from mice transplanted with A375, HCC364 and Colo-205 cell lines, respectively. The first row illustrates the effect of apatinib on the body weight of mice. The second and the third rows illustrated the effect of apatinib on the tumor sizes (n=6). **Figure S2.** Immunohistochemistry analyses based on cellular proteins. A, B, and C are A375, HCC364, and Colo-205 cell protein assay data, respectively. The above figure shows the relative optical density of each group of cells. The figure below shows the target band after exposure of the target protein. The degree of protein phosphorylation is expressed as the ratio of phosphorylated protein to total protein. Three biological replicates (n=3) were set in the experiment, and the data were expressed as mean ± SD, ^*^P<0.05, ^**^P<0.01 vs. cell control group. ① control group, ② vemurafenib control group, ③ apatinib treatment group (10 nM); ④ apatinib treatment group (20 nM), ⑤ apatinib treatment group (50 nM). **Figure S3.** Beneficial effect of apatinib HCC364 mice and possible cellular mechanism. Inhibition of apatinib on tumor tissue signaling pathway in HCC364 nude mice; the figure above shows the comparison between the groups of protein phosphorylation rates; the figure below shows the scan of the target bands. 6 nude mice per group (n=6), the data is expressed as ^*^P < 0.05, ^**^P < 0.01 (vs. vehicle group).

## Data Availability

The datasets used and analyzed during the current study are available from the corresponding author on reasonable request.

## Abbreviations

NSCLC: Non-small-cell lung cancer
RR: Response rate
VEGFR2: Vascular endothelial growth factor receptor-2
