# Supplementary material for: In vitro and in vivo analyses on anti-NSCLC activity of apatinib: rediscovery of a new drug target V600E mutation
Source: Cancer Cell Int. 2023 Feb 9;23:21. doi: 10.1186/s12935-022-02723-7 (PMC9909954; doi:10.1186/s12935-022-02723-7)
Supplement: Supplementary file 1 — Additional file 1: Table S1. The inhibitory activity data of apatinib against each kinase. [file 12935_2022_2723_MOESM1_ESM.docx]

**Table S1 The inhibitory activity data of apatinib against each kinase**

| **Number** | **Project** | **Test Compound Name** | **Test Compound Concentration (nM)** | **Kinase Tested** | **Inhibitory rate (%)** | **Kinase Part/Lot** |
| --- | --- | --- | --- | --- | --- | --- |
| 1 | SSBK12211_35390 | Apatinib | 100 | KIT Y823D | 99 | PV6322/1693286 |
| 2 | SSBK12211_35390 | Apatinib | 100 | CSF1R (FMS) | 73 | PV3249/1510605 |
| 3 | SSBK12211_35390 | Apatinib | 100 | RET G691S | 73 | PV6214/1570982 |
| 4 | SSBK12211_35390 | Apatinib | 100 | KIT V559D T670I | 70 | PV6316/1579472 |
| 5 | SSBK12211_35390 | Apatinib | 100 | RET M918T | 67 | PV6217/1570989 |
| 6 | SSBK12211_35390 | Apatinib | 100 | PDGFRA V561D | 64 | PV4680/1240446 |
| 7 | SSBK12211_35390 | Apatinib | 100 | KDR (VEGFR2) | 61 | PV3660/1391241 |
| 8 | SSBK12211_35390 | Apatinib | 100 | KIT N822K | 59 | PV6310/1576886 |
| 9 | SSBK12211_35390 | Apatinib | 100 | RAF1 (cRAF) Y340D Y341D | 57 | PV3805/1524194 |
| 10 | SSBK12211_35390 | Apatinib | 100 | FLT4 (VEGFR3) | 56 | PV4129/38454 |
| 11 | SSBK12211_35390 | Apatinib | 100 | BRAF V600E | 51 | PV3849/910409 |
| 12 | SSBK12211_35390 | Apatinib | 100 | RET Y791F | 50 | PV4396/36639 |
| 13 | SSBK12211_35390 | Apatinib | 100 | FLT1 (VEGFR1) | 48 | PV3666/1449776 |
| 14 | SSBK12211_35390 | Apatinib | 100 | BRAF | 43 | PV3848/1258788 |
| 15 | SSBK12211_35390 | Apatinib | 100 | LYN A | 40 | PV6448/1740264 |
| 16 | SSBK12211_35390 | Apatinib | 100 | EGFR (ErbB1) L861Q | 35 | PV3873/34562 |
| 17 | SSBK12211_35390 | Apatinib | 100 | KIT T670I | 34 | PV3869/34504 |
| 18 | SSBK12211_35390 | Apatinib | 100 | RET | 33 | PV3819/853376 |
| 19 | SSBK12211_35390 | Apatinib | 100 | FLT3 | 33 | PV3182/1614759 |
| 20 | SSBK12211_35390 | Apatinib | 100 | LYN B | 33 | P2907/1412850 |
| 21 | SSBK12211_35390 | Apatinib | 100 | KIT D820E | 31 | PV6307/1576885 |
| 22 | SSBK12211_35390 | Apatinib | 100 | EGFR (ErbB1) | 30 | PV3872/1489094 |
| 23 | SSBK12211_35390 | Apatinib | 100 | PDGFRB (PDGFR beta) | 25 | P3082/27567 |
| 24 | SSBK12211_35390 | Apatinib | 100 | FGFR3 K650E | 25 | PV4392/36445 |
| 25 | SSBK12211_35390 | Apatinib | 100 | EGFR (ErbB1) d746-750 | 24 | PV6178/1737746 |
| 26 | SSBK12211_35390 | Apatinib | 100 | KIT D816H | 21 | PV6196/1570980 |
| 27 | SSBK12211_35390 | Apatinib | 100 | MUSK | 20 | PV3834/1671194 |
| 28 | SSBK12211_35390 | Apatinib | 100 | KIT | 20 | P3081/1559213 |
| 29 | SSBK12211_35390 | Apatinib | 100 | NTRK1 (TRKA) | 20 | PV3144/1606305 |
| 30 | SSBK12211_35390 | Apatinib | 100 | YES1 | 19 | A15557/1383140 |
| 31 | SSBK12211_35390 | Apatinib | 100 | FLT3 ITD | 18 | PV6190/1641975 |
| 32 | SSBK12211_35390 | Apatinib | 100 | PDGFRA D842V | 17 | PV4203/269691 |
| 33 | SSBK12211_35390 | Apatinib | 100 | ROS1 | 16 | PV3814/846951 |
| 34 | SSBK12211_35390 | Apatinib | 100 | TEK (TIE2) R849W | 16 | PV6226/1640777 |
| 35 | SSBK12211_35390 | Apatinib | 100 | IGF1R | 15 | PV3250/1681148 |
| 36 | SSBK12211_35390 | Apatinib | 100 | ALK L1196M | 14 | PV6166/1636937 |
| 37 | SSBK12211_35390 | Apatinib | 100 | HCK | 14 | PV6128/862448 |
| 38 | SSBK12211_35390 | Apatinib | 100 | SRC N1 | 13 | P2904/21068 |
| 39 | SSBK12211_35390 | Apatinib | 100 | FYN | 13 | P3042/1265860 |
| 40 | SSBK12211_35390 | Apatinib | 100 | ERBB4 (HER4) | 12 | PV3626/32657 |
| 41 | SSBK12211_35390 | Apatinib | 100 | KIT V654A | 12 | PV4132/35129 |
| 42 | SSBK12211_35390 | Apatinib | 100 | EPHA6 | 11 | PV6337/1754255 |
| 43 | SSBK12211_35390 | Apatinib | 100 | FLT3 D835Y | 11 | PV3967/1566961 |
| 44 | SSBK12211_35390 | Apatinib | 100 | LCK | 11 | P3043/1475041 |
| 45 | SSBK12211_35390 | Apatinib | 100 | EPHB2 | 10 | PV3625/1386867 |
| 46 | SSBK12211_35390 | Apatinib | 100 | SRC | 10 | P3044/1255538 |
| 47 | SSBK12211_35390 | Apatinib | 100 | SRMS (Srm) | 10 | PV4214/306354 |
| 48 | SSBK12211_35390 | Apatinib | 100 | AKT1 (PKB alpha) | 9 | P2999/1629728 |
| 49 | SSBK12211_35390 | Apatinib | 100 | EPHA5 | 9 | PV3840/34383 |
| 50 | SSBK12211_35390 | Apatinib | 100 | EPHA2 | 9 | PV3688/1144599 |
| 51 | SSBK12211_35390 | Apatinib | 100 | ABL1 G250E | 8 | PV3865/34529 |
| 52 | SSBK12211_35390 | Apatinib | 100 | PTK2 (FAK) | 8 | PV3832/1646492 |
| 53 | SSBK12211_35390 | Apatinib | 100 | ABL1 H396P | 8 | PV6148/1570966 |
| 54 | SSBK12211_35390 | Apatinib | 100 | KIT A829P | 8 | PV6193/1570981 |
| 55 | SSBK12211_35390 | Apatinib | 100 | ABL1 M351T | 8 | PV6151/1636303 |
| 56 | SSBK12211_35390 | Apatinib | 100 | PIK3C2A (PI3K-C2 alpha) | 7 | PV5586/1405300 |
| 57 | SSBK12211_35390 | Apatinib | 100 | MERTK (cMER) | 7 | PV3627/1691294 |
| 58 | SSBK12211_35390 | Apatinib | 100 | ZAP70 | 7 | P2782/1703735 |
| 59 | SSBK12211_35390 | Apatinib | 100 | MATK (HYL) | 7 | PV3370/31553 |
| 60 | SSBK12211_35390 | Apatinib | 100 | PIK3C2B (PI3K-C2 beta) | 7 | PV5374/1766082 |
| 61 | SSBK12211_35390 | Apatinib | 100 | FYN A | 7 | PV6346/1639434 |
| 62 | SSBK12211_35390 | Apatinib | 100 | DDR2 N456S | 7 | PV6172/1626057 |
| 63 | SSBK12211_35390 | Apatinib | 100 | INSR | 7 | PV3781/1378058 |
| 64 | SSBK12211_35390 | Apatinib | 100 | INSRR (IRR) | 7 | PV3808/34272 |
| 65 | SSBK12211_35390 | Apatinib | 100 | FRK (PTK5) | 7 | PV3874/1209097 |
| 66 | SSBK12211_35390 | Apatinib | 100 | ALK F1174L | 7 | PV6160/1636936 |
| 67 | SSBK12211_35390 | Apatinib | 100 | AKT2 (PKB beta) | 6 | PV3184/28770 |
| 68 | SSBK12211_35390 | Apatinib | 100 | CSK | 6 | P2927/1391666 |
| 69 | SSBK12211_35390 | Apatinib | 100 | SYK | 6 | PV3857/756818 |
| 70 | SSBK12211_35390 | Apatinib | 100 | KIT D816V | 6 | PV6199/1570986 |
| 71 | SSBK12211_35390 | Apatinib | 100 | ITK | 5 | PV3875/1736055 |
| 72 | SSBK12211_35390 | Apatinib | 100 | FRAP1 (mTOR) | 5 | PV4753/1739838 |
| 73 | SSBK12211_35390 | Apatinib | 100 | AXL R499C | 5 | PV6253/1578673 |
| 74 | SSBK12211_35390 | Apatinib | 100 | ABL1 T315I | 5 | PV3866/39639 |
| 75 | SSBK12211_35390 | Apatinib | 100 | RET V804L | 5 | PV4397/36640 |
| 76 | SSBK12211_35390 | Apatinib | 100 | EGFR (ErbB1) L858R | 5 | PV4128/853375 |
| 77 | SSBK12211_35390 | Apatinib | 100 | EPHB3 | 4 | PV3658/33066 |
| 78 | SSBK12211_35390 | Apatinib | 100 | PDGFRA (PDGFR alpha) | 4 | PV3811/1269727 |
| 79 | SSBK12211_35390 | Apatinib | 100 | ABL2 (Arg) | 4 | PV3266/850069 |
| 80 | SSBK12211_35390 | Apatinib | 100 | AXL | 4 | PV3971/1653016 |
| 81 | SSBK12211_35390 | Apatinib | 100 | ABL1 Y253F | 4 | PV3863/34531 |
| 82 | SSBK12211_35390 | Apatinib | 100 | TXK | 4 | PV5860/750657 |
| 83 | SSBK12211_35390 | Apatinib | 100 | ALK C1156Y | 3 | PV6157/1570963 |
| 84 | SSBK12211_35390 | Apatinib | 100 | JAK2 JH1 JH2 V617F | 3 | PV4336/463344 |
| 85 | SSBK12211_35390 | Apatinib | 100 | TEK (Tie2) | 3 | PV3628/34398 |
| 86 | SSBK12211_35390 | Apatinib | 100 | BMX | 3 | PV3371/1600039 |
| 87 | SSBK12211_35390 | Apatinib | 100 | LTK (TYK1) | 3 | PV4651/768522 |
| 88 | SSBK12211_35390 | Apatinib | 100 | KIT T670E | 3 | PV6313/1575536 |
| 89 | SSBK12211_35390 | Apatinib | 100 | FGFR3 K650M | 3 | PV6187/1573650 |
| 90 | SSBK12211_35390 | Apatinib | 100 | ABL1 E255K | 3 | PV3864/34528 |
| 91 | SSBK12211_35390 | Apatinib | 100 | TEK (TIE2) Y1108F | 3 | PV6229/1629376 |
| 92 | SSBK12211_35390 | Apatinib | 100 | MERTK (cMER) A708S | 3 | PV6325/1578675 |
| 93 | SSBK12211_35390 | Apatinib | 100 | ABL1 Q252H | 3 | PV6154/1627756 |
| 94 | SSBK12211_35390 | Apatinib | 100 | EGFR (ErbB1) T790M L858R | 2 | PV4879/1498821 |
| 95 | SSBK12211_35390 | Apatinib | 100 | ALK R1275Q | 2 | PV6169/1570970 |
| 96 | SSBK12211_35390 | Apatinib | 100 | EPHA1 | 2 | PV3841/1531110 |
| 97 | SSBK12211_35390 | Apatinib | 100 | TNK2 (ACK) | 1 | PV4807/407338 |
| 98 | SSBK12211_35390 | Apatinib | 100 | ERBB2 (HER2) | 1 | PV3366/1320117 |
| 99 | SSBK12211_35390 | Apatinib | 100 | DDR2 T654M | 1 | PV6175/1716840 |
| 100 | SSBK12211_35390 | Apatinib | 100 | EPHA7 | 1 | PV3689/33790 |
| 101 | SSBK12211_35390 | Apatinib | 100 | NTRK2 (TRKB) | 1 | PV3616/35706 |
| 102 | SSBK12211_35390 | Apatinib | 100 | EGFR (ErbB1) T790M | 1 | PV4803/1229180 |
| 103 | SSBK12211_35390 | Apatinib | 100 | TYRO3 (RSE) | 1 | PV3828/1633300 |
| 104 | SSBK12211_35390 | Apatinib | 100 | EPHA4 | 0 | PV3651/32933 |
| 105 | SSBK12211_35390 | Apatinib | 100 | EPHB1 | 0 | PV3786/34225 |
| 106 | SSBK12211_35390 | Apatinib | 100 | FGR | 0 | P3041/1226633 |
| 107 | SSBK12211_35390 | Apatinib | 100 | EPHA3 | 0 | PV3359/30916 |
| 108 | SSBK12211_35390 | Apatinib | 100 | FGFR3 G697C | 0 | PV6184/1570969 |
| 109 | SSBK12211_35390 | Apatinib | 100 | ABL1 | 0 | P3049/1258786 |
| 110 | SSBK12211_35390 | Apatinib | 100 | AKT3 (PKB gamma) | 0 | PV3185/28771 |
| 111 | SSBK12211_35390 | Apatinib | 100 | MST1R (RON) | -1 | PV4314/765277 |
| 112 | SSBK12211_35390 | Apatinib | 100 | FGFR4 | -1 | P3054/26967 |
| 113 | SSBK12211_35390 | Apatinib | 100 | RET V804M | -2 | PV6223/1697905 |
| 114 | SSBK12211_35390 | Apatinib | 100 | DDR2 | -2 | PV3870/933644 |
| 115 | SSBK12211_35390 | Apatinib | 100 | EPHA8 | -2 | PV3844/36870 |
| 116 | SSBK12211_35390 | Apatinib | 100 | EPHB4 | -2 | PV3251/1311417 |
| 117 | SSBK12211_35390 | Apatinib | 100 | MET D1228H | -2 | PV6208/1570985 |
| 118 | SSBK12211_35390 | Apatinib | 100 | TEC | -3 | PV3269/910411 |
| 119 | SSBK12211_35390 | Apatinib | 100 | ALK | -3 | PV3867/1542512 |
| 120 | SSBK12211_35390 | Apatinib | 100 | NTRK3 (TRKC) | -3 | PV3617/708766 |
| 121 | SSBK12211_35390 | Apatinib | 100 | PDGFRA T674I | -4 | PV3847/1612545 |
| 122 | SSBK12211_35390 | Apatinib | 100 | TYK2 | -4 | PV4790/1681147 |
| 123 | SSBK12211_35390 | Apatinib | 100 | PTK2B (FAK2) | -4 | PV4567/883370 |
| 124 | SSBK12211_35390 | Apatinib | 100 | MET M1250T | -4 | PV3968/34718 |
| 125 | SSBK12211_35390 | Apatinib | 100 | PTK6 (Brk) | -5 | PV3291/1611016 |
| 126 | SSBK12211_35390 | Apatinib | 100 | BTK | -5 | PV3363/1493953 |
| 127 | SSBK12211_35390 | Apatinib | 100 | FGFR1 V561M | -6 | PV6343/1578678 |
| 128 | SSBK12211_35390 | Apatinib | 100 | FGFR1 | -7 | PV3146/1559215 |
| 129 | SSBK12211_35390 | Apatinib | 100 | BLK | -7 | PV3683/33635 |
| 130 | SSBK12211_35390 | Apatinib | 100 | JAK2 JH1 JH2 | -7 | PV4393/311662 |
| 131 | SSBK12211_35390 | Apatinib | 100 | FER | -8 | PV3806/38946 |
| 132 | SSBK12211_35390 | Apatinib | 100 | FGFR3 | -8 | PV3145/28459 |
| 133 | SSBK12211_35390 | Apatinib | 100 | JAK3 | -9 | PV3855/1386865 |
| 134 | SSBK12211_35390 | Apatinib | 100 | JAK1 | -9 | PV4774/1619716 |
| 135 | SSBK12211_35390 | Apatinib | 100 | FES (FPS) | -10 | PV3354/35734 |
| 136 | SSBK12211_35390 | Apatinib | 100 | FGFR2 | -11 | PV3368/31517 |
| 137 | SSBK12211_35390 | Apatinib | 100 | MET (cMet) | -12 | PV3143/722459 |
| 138 | SSBK12211_35390 | Apatinib | 100 | JAK2 | -20 | PV4210/36301 |
