# Supplementary material for: In vitro and in vivo analyses on anti-NSCLC activity of apatinib: rediscovery of a new drug target V600E mutation
Source: Cancer Cell Int. 2023 Feb 9;23:21. doi: 10.1186/s12935-022-02723-7 (PMC9909954; doi:10.1186/s12935-022-02723-7)

**Supplementary Figures**

**
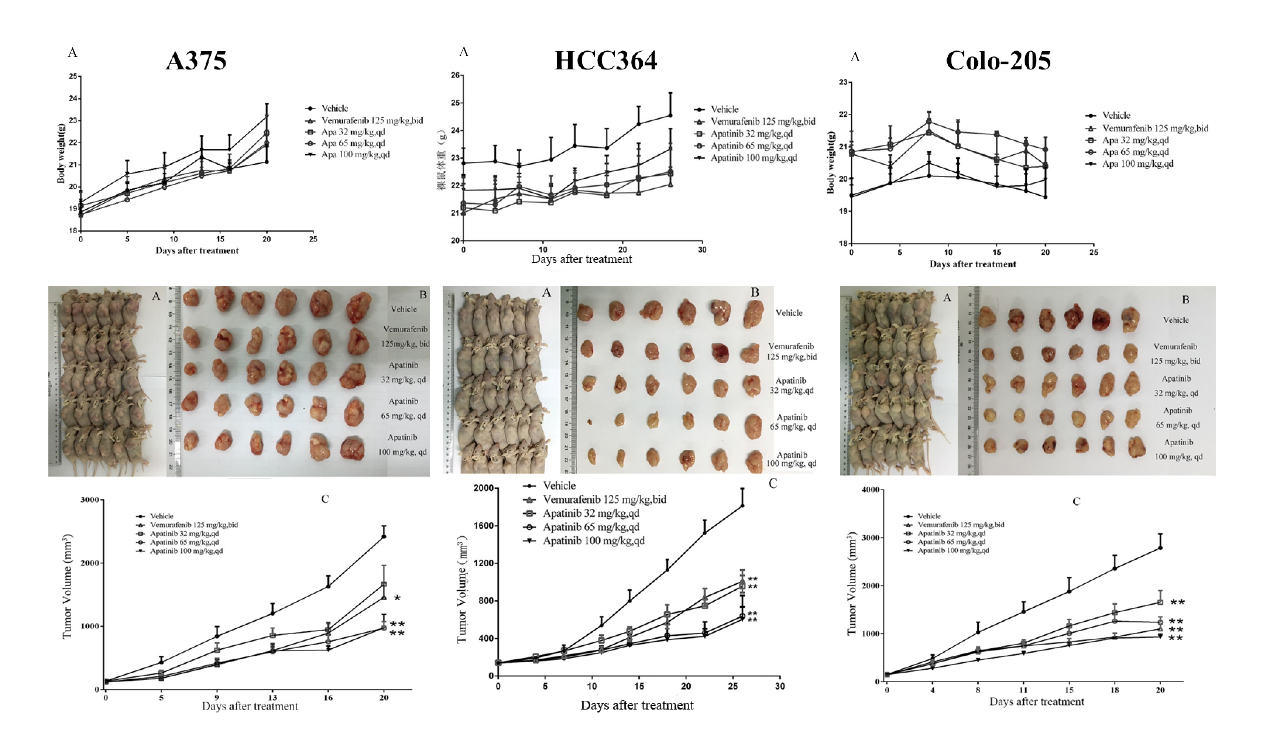
**

**Fig. S1 Tumor suppression effects of apatinib.** From left to right, data were acquired from mice transplanted with A375, HCC364 and Colo-205 cell lines, respectively. The first row illustrates the effect of apatinib on the body weight of mice. The second and the third rows illustrated the effect of apatinib on the tumor sizes (n=6).

**
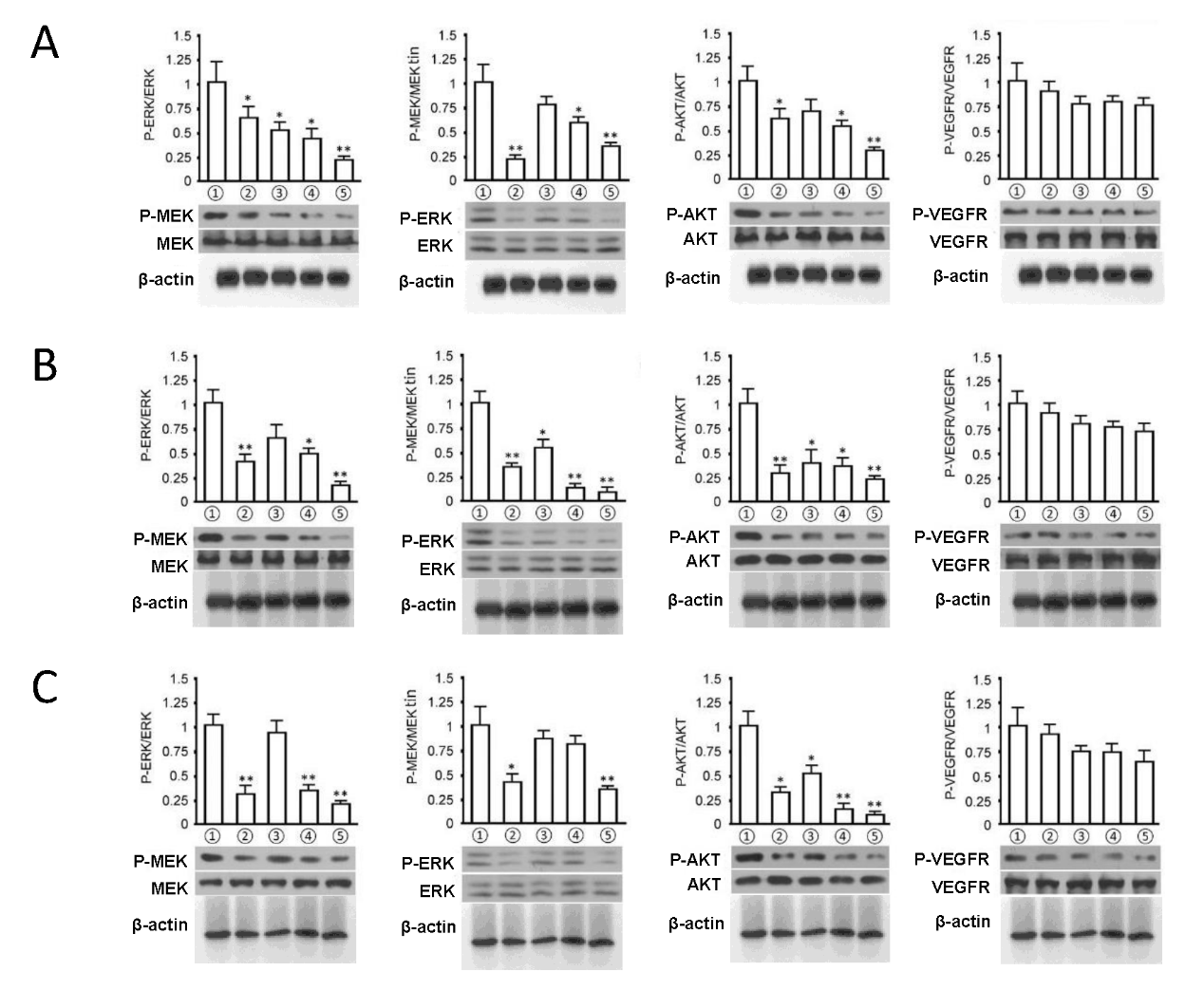
**

**Fig. S2 Immunohistochemistry analyses based on cellular proteins.** A, B, and C are A375, HCC364, and Colo-205 cell protein assay data, respectively. The above figure shows the relative optical density of each group of cells. The figure below shows the target band after exposure of the target protein. The degree of protein phosphorylation is expressed as the ratio of phosphorylated protein to total protein. Three biological replicates (n=3) were set in the experiment, and the data were expressed as mean ± SD, ^*^P<0.05 ^**^P<0.01 vs. cell control group. ① control group, ② vemurafenib control group, ③ apatinib treatment group (10 nM); ④ apatinib treatment group (20 nM), ⑤ apatinib treatment group (50 nM).

**Fig. S3 Beneficial effect of apatinib HCC364 mice and possible cellular mechanism.** Inhibition of apatinib on tumor tissue signaling pathway in HCC364 nude mice; the figure above shows the comparison between the groups of protein phosphorylation rates; the figure below shows the scan of the target bands. 6 nude mice per group (n=6), the data is expressed as ^*^P < 0.05, ^**^P < 0.01 (vs. vehicle group).


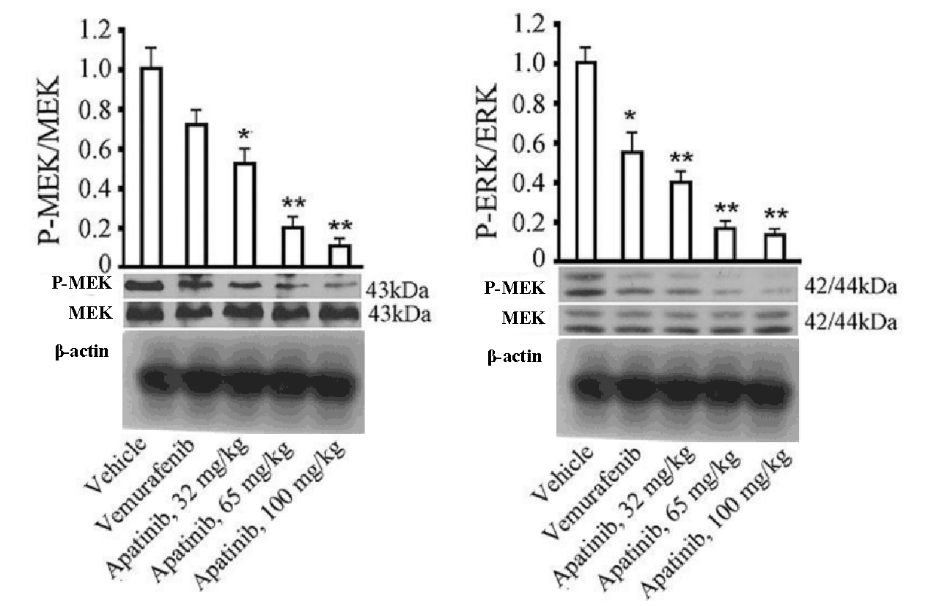

Supplement: Supplementary file 2 — Additional file 2: Figure S1. Tumor suppression effects of apatinib. From left to right, data were acquired from mice transplanted with A375, HCC364 and Colo-205 cell lines, respectively. The first row illustrates the effect of apatinib on the body weight of mice. The second and the third rows illustrated the effect of apatinib on the tumor sizes (n=6). Figure S2. Immunohistochemistry analyses based on cellular proteins. A, B, and C are A375, HCC364, and Colo-205 cell protein assay data, respectively. The above figure shows the relative optical density of each group of cells. The figure below shows the target band after exposure of the target protein. The degree of protein phosphorylation is expressed as the ratio of phosphorylated protein to total protein. Three biological replicates (n=3) were set in the experiment, and the data were expressed as mean ± SD, *P<0.05, **P<0.01 vs. cell control group. ① control group, ② vemurafenib control group, ③ apatinib treatment group (10 nM); ④ apatinib treatment group (20 nM), ⑤ apatinib treatment group (50 nM). Figure S3. Beneficial effect of apatinib HCC364 mice and possible cellular mechanism. Inhibition of apatinib on tumor tissue signaling pathway in HCC364 nude mice; the figure above shows the comparison between the groups of protein phosphorylation rates; the figure below shows the scan of the target bands. 6 nude mice per group (n=6), the data is expressed as *P < 0.05, **P < 0.01 (vs. vehicle group). [file 12935_2022_2723_MOESM2_ESM.docx]
